# Swipe & Slice: Decoding Digital Struggles with NSSI in Young Italians

**DOI:** 10.1192/j.eurpsy.2024.376

**Published:** 2024-08-27

**Authors:** S. Reina, G. Longo, L. Orsolini, U. Volpe

**Affiliations:** University Psychiatric Clinic, Department of Clinical Neuroscience/DIMSC, Università Politecnica delle Marche, Ancona, Italy

## Abstract

**Introduction:**

Non-suicidal self-injury (NSSI) is defined as any deliberate destruction of one’s body tissue, engaged in for reasons that are non-suicidal. Online platforms, notably social media, witness a surge in NSSI-related content, amplified by the COVID-19 pandemic. Young individuals increase video and post uploads, prompting scholarly inquiry into the impact on vulnerable demographics in the online environment. Despite potential benefits, concerns surface regarding content reinforcing self-injurious behavior. The Blue Whale phenomenon exemplifies serious consequences in this digital landscape.

**Objectives:**

The present study aims at screening the prevalence of NSSIs on SNS among Italian young people.

**Methods:**

An observational cross-sectional study was conducted by recruiting 373 Italian young people (aged 18-25). Bergen Social Media Addiction Scale (BSMAS), Fear Of Missing Out Scale (FOMO), Inventory of Statements About Self-Injury (ISAS) were administered to investigate the relationship between NSSIs, social media use and frequency and underpinned motivations.

**Results:**

Overall, 99.7 % (n=372) of participants declared to have used at least one social network. Around 92.5 % (n=345) declared to know Blue Whale Challenge and more than half of the sample (51.5%) referred to have looked for NSSI contents on SNS, mostly (28.7 % (n=107)) have sought for curiosity, 17.7 % (n=66) have sought for help/support. 53.4 % (n=199) of the sample was found to have problematic social media use (PSMU) according to BSMAS. 85 % (n=317) have committed self-injurious gestures in the past, 66.2 % (n=247) practice NSSI currently, most subjects practice them to vent 51.7% (n=193), calm themselves 41.6% (n=155), and punish themselves 30% (n=112). The mean age of transgender and nonbinary subjects (30 % n=112)) who sought/saw content pertaining to NSSIs appears to be lower (p=0.033) than cisgender subjects. Those who searched for content inherent to NSSIs scored higher mean scores on the FOMO (p=0.022) and BSMAS (p=0.013) scales. Those who follow social pages inherent to NSSIs scored higher on the FOMO scale (p=0.035). Subjects who practice NSSIs at their present state, on average, have higher scores on the FOMO and BSMAS scales (p=<.001). Linear regression analysis was conducted showing an association between BSMAS and FOMO (R²=0.199, B=0.260; F(1.371)=92.334; p=<.001). Logistic regression analyses were conducted to define the effects of FOMO, PMSU, sex, and NSSI search on the development of self-injurious conduct. The logistic regression model was statistically significant, χ2 (1)=3.909; p=0.048.

**Conclusions:**

The study examines NSSI behaviors among young Italian college students on digital platforms, particularly social networks. It stresses the critical need for targeted interventions, addressing concerns like social media addiction, to provide essential mental health support and foster a safer online environment for this population.

**Disclosure of Interest:**

None Declared

